# Nomogram to Predict the Probability of Functional Dependence in Early Parkinson’s Disease

**DOI:** 10.3233/JPD-223501

**Published:** 2023-01-31

**Authors:** Dora Valent, Florian Krismer, Anna Grossauer, Marina Peball, Beatrice Heim, Philipp Mahlknecht, Atbin Djamshidian, Werner Poewe, Klaus Seppi

**Affiliations:** Department of Neurology, Medical University of Innsbruck, Innsbruck, Austria

**Keywords:** Parkinson’s disease, nomograms, functional status, prognosis, risk, early diagnosis

## Abstract

**Background:**

Early identification of Parkinson’s disease (PD) patients at risk for becoming functionally dependent is important for patient counseling. Several models describing the relationship between predictors and outcome have been reported, however, most of these require computer software for practical use.

**Objective:**

Here we report the development of a risk nomogram allowing an approximate graphical computation of the risk of becoming functionally dependent in early PD.

**Methods:**

We analyzed data form the Parkinson’s Progression Markers Initiative cohort of newly diagnosed PD patients from baseline through the first 5 years of follow-up. Functional dependence was defined as a score < 80 on the Schwab & England Activities of Daily Living scale. A binary logistic model was developed to estimate the risk of functional dependence and based on the results, a nomogram for the prediction of functional dependence was drawn in order to provide an easy-to-use tool in clinical and academic settings as a part of personalized medicine approach to PD treatment.

**Results:**

At baseline, three patients and over the five-year follow-up, 85 (22%) out of 395 patients were functionally dependent as scored by the Schwab & England Activities of Daily Living rating scale. The binary logistic model showed that clinical parameters such as MDS-UPDRS I (rater part), MDS-UPDRS II, and MDS-UPDRS axial motor score were significant predictors for functional dependence within 5 years.

**Conclusion:**

We here provide an easy-to-use tool to estimate the risk of functional dependence in PD patients based on the MDS-UPDRS part I, II and axial motor score.

## INTRODUCTION

Parkinson’s disease (PD) follows heterogeneous trajectories of clinical progression of its motor and non-motor symptoms that may lead to loss of functional independence, reduced quality of life as well as familial and societal burden [1]. Due to marked inter-individual differences in symptom progression predicting the timeframe of onset of functional disabilities is difficult at an individual patient level. On the other hand, such information is of major concern to patients and of obvious importance in personalized patient care, as well as for designing clinical studies.

Previous reports have identified age, disease duration, the Movement Disorders Society-Unified Parkinson’s Disease Rating Scale (MDS-UPDRS) motor score, its axial motor subscore, total MDS-UPDRS score, UPDRS Activities of Daily Living (ADL) score, cognitive problems and daytime fatigue, among others, to be independent predictors of disability [2–8]. Studies addressing the progressive loss of functional independence in PD have been limited by overall poor data quality, incongruity in study populations and methodological differences including different definitions of functional dependency [7].

In recent years, several authors have highlighted the need for a tool that may be used to integrate recognized biomarkers and aid treatment planning in light of more and more emerging pieces in the etiology of PD [9–11]. A nomogram is a unique graphical calculator designed to include continuous or categorical variables where each point value is assigned prognostic importance. It can be based on any function such as Cox hazard ratio regression models or logistic regression [12]. Nomograms are an accepted standard tool in cancer treatment to predict survival and have started to make their way into movement disorder research as well [13]. We sought to develop the first nomogram to calculate the probability of functional dependence in PD patients.

The aim of this study was to quantify risk factors associated with the progression of dependence for an individual patient with PD and predict the probability of reaching functional dependence within 5 years after diagnosis. The integrative nomogram we developed aims to assist decision-making and timely therapeutic acquisition/intervention at the time of disease onset in patients with PD.

## METHODS

### Participants

For these analyses we used data of 395 PD patients obtained from the Parkinson’s Progression Marker Initiative (PPMI), a publicly-available, longitudinal, prospective database of over 400 patients with early stage PD [14]. The homepage (https://www.ppmi-info.org) gives full information about the PPMI study, including inclusion and exclusion criteria, recruitment strategy, study sites, full list of assessments and study related procedures. Study participants underwent regular standardized and validated evaluations, clinical and cognitive assessments, imaging examinations, and biological sampling at 3-month intervals in the first year and 6-month intervals in subsequent years. Inclusion criteria requirements for patients with PD included symptoms of rest tremor, bradykinesia, or rigidity, a Hoehn & Yahr stage I or II at enrollment, and a diagnosis of PD for less than 2 years at screening. Eligible PD patients had neuroimaging findings on dopamine transporter (DaT)-spectral positron emission computed tomography (SPECT) (DaTSCAN) that supported the PD diagnosis. Each participating PPMI site received approval from an ethical standards committee on human experimentation before study initiation and written informed consent for research was obtained from all participants in the study. Baseline visit equals to time of diagnoses.

### Assessments

The Schwab and England (S&E) scale is a validated and recommended tool to assess functional disability in PD [15]. Similar to previous studies we used a score of < 80% at any follow-up visit within the first 5 years after enrollment into the PPMI as an anchor for functional dependence [2, 6, 16].

The following PPMI assessments were considered as predictors of functional dependence: older age at diagnosis [2, 5, 7, 8], more severe axial impairment on the MDS-UPDRS [17–21], worse cognition (lower MoCA score) [2–5], worse REM Sleep Behavior Disorder Screening Questionnaire (RBDSQ score) [22], fatigue (MDS-UPDRS item 1.13) [3, 8], bradykinesia sub-score from MDS-UPDRS [7, 22], comorbidities measured by the Charlson Comorbidity Index [23], and sex [3, 24]. Additionally, we assessed the following predictors: autonomic system dysfunction (SCOPA-AUT), Epworth Sleepiness Scale, CSF biomarkers (Aβ_42_, pTau, tTau, mtDNA deletion, nDNA B2M-CN, α-Synuclein, GFAP, interleukin-6, s100, Neurofilament light chain, soluble TREM2, and YKL-40), MDS-UPDRS Part I, Part II, Part III total, and DaT Scan caudate and putamen density.

We used MDS-UPDRS items: 3.1 speech, 3.2 facial expression, 3.3a neck rigidity, 3.9 arising from chair, 3.10 gait, 3.11 freezing of gait, 3.12 postural stability, 3.13 posture, and 3.14 body bradykinesia to build a composite axial motor score to include all axial symptoms [19].

### Statistics

Data used in the preparation of this article were obtained from the Parkinson’s Progression Markers Initiative (PPMI) database (https://www.ppmi-info.org/data). For up-to-date information on the study, visit www.ppmi-info.org. Data were accessed on 25 November, 2021. In 2021, PPMI participants were assigned to cohorts and subgroups in an analytic dataset, based on a central review of the most recent longitudinal data. The analytic data set cohort assignments were used for all data analysis.

R software (version 4.1.2; R Foundation for Statistical Computing, Vienna, Austria) was used for statistical analyses. The significance level was set at *p* < 0.05. Variables with more than 20% missing values were excluded from further data analysis. The remaining missing values were imputed using nearest neighbors method (Gower’s distance was used to determine the five nearest neighbors; once the nearest neighbors were determined, the mode was used to predict nominal variables and the mean was used for numeric data). The dataset was randomly split into a discovery (80%) and a testing dataset (20%). Predictors of functional dependence in PD were determined in the discovery cohort by a L1-regularized logistic regression model implementing the least absolute shrinkage and selection operator (LASSO). A pre-defined set of model tuning parameters was evaluated across bootstrap resamples and the tuning parameter combination with the best performance value (i.e., the largest area under the receiver-operating characteristics curve) was selected. Overall, model performance was evaluated in the test cohort. The nomogram was developed on the entire dataset exploiting a binary logistic regression model including all predictors identified in the previous step.

## RESULTS

### Clinical characteristics of patients

The demographic characteristics and clinical data of PD patients are summarized in Table 1. Total number of patients included was 395, out of which 259 were male and 136 female. There was a significant decrease in the S&E score from V0 to V12/Year 5 when considering the patients who were functionally dependent (BL (n)=3, Y5 (n)=40). The total number of patients who were rated an S&E score of < 80 at any time during the five years were in total 85, however, 45 of these patients dropped out, died or improved through medication therapy through the 5^th^ year visit. At Year 5, the frequency of functional dependency was 13.3 times compared to Baseline. For the patients who were categorized as functionally dependent within 5 years, the median baseline total MDS-UPDRS I score was 1 (IQR 0-3, *p* < 0.001), the baseline MDS-UPDRS II score was 8 (IQR 5-11, *p* < 0.001) and baseline axial motor score was 7 (IQR 5-8, *p* < 0.001). In comparison, patients who were functionally independent within the 5 years of analysis had lower median baseline scores or a narrower range, namely, an MDS-UPDRS part I score of 1 (IQR 0-2), an MDS-UPDRS part II score was 4 (IQR 2-7), and an axial motor score was 4 (IQR 3-7).

**Table 1:** Demographical data. Table 1 shows demographical data and frequencies of clinical predictors by functional dependency within year 5 (Visit 12)

Characteristic	Functional Independent^1^ *n* = 310	Functional Dependent^1^ *n* = 85	p^2^
**Sex**			0.5
**Female**	104 (34%)	32 (38%)
**Male**	206 (66%)	53 (62%)
**Age (at BL)**	62 (55, 69); 61±10	64 (56, 70); 63±10	0.15
**S&E (at BL)**	95 (90,100); 94±5	90 (90,95); 90±7	<0.001
**SCOPA sum**	8 (5, 12); 9±5.9	10.0 (7, 15); 11.5±6.5	<0.001
**Categorical RBDSQ**	69 (22%)	29 (35%)	0.023
**MDS-UPDRS axial**	4 (3, 7); 4.82±2.65	7 (5, 8); 6.42±2.67	<0.001
**MDS-UPDRS I total**	1 (0, 2); 1.05±1.47	1 (0, 3); 1.81±1.75	<0.001
**MDS-UPDRS II total**	4 (2, 7); 5.2±3.8	8 (5, 11); 8.3±4.7	<0.001
**Charlson Comorbidity index**			0.2
**0**	247 (80%)	63 (74%)
**1**	39 (13%)	13 (15%)
**2**	17 (5.5%)	3 (3.5%)
**3**	5 (1.6%)	4 (4.7%)
**4**	1 (0.3%)	1 (1.2%)
**5**	0 (0%)	1 (1.2%)
**6**	0 (0%)	0 (0%)
**7**	1 (0.3%)	0 (0%)

^1^Median (IQR); Mean±standard deviation; n (%). ^2^Wilcoxon rank sum test; Pearson’s Chi-squared test; Fisher’s exact test. BL, Baseline; S&E, Schwab & England; SCOPA, SCales for Outcomes in PArkinson’s disease; RBD, REM Sleep Behavior Screening Questionnaire; MDS-UPDRS, Movement Disorder Society- Unified Parkinson’s Disease Rating Scale.

### Lasso model

**Fig. 1:**
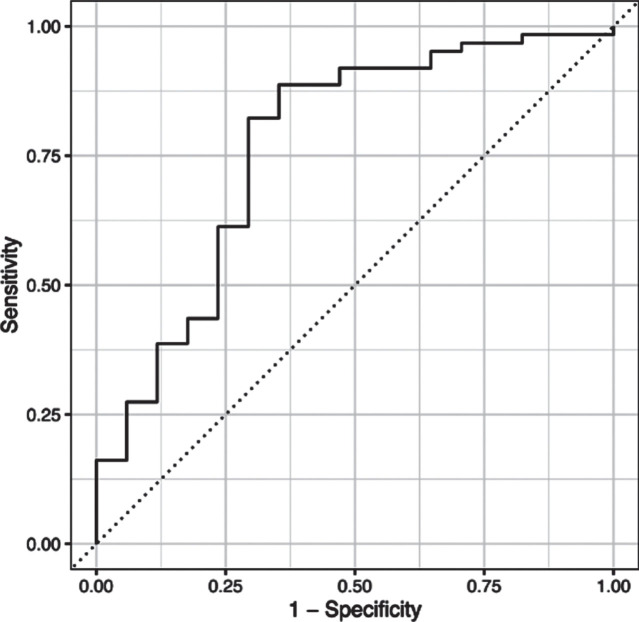
ROC curve analysis. The combination of the three predictors (MDS-UPDRS I Rater, MDS-UPDRS II, and axial motor score) yielded an overall accuracy of 78.5% and the area under the receiver-operating characteristics curve was 76.4%.

L1-regularized logistic regression model implementing the least absolute shrinkage and selection operator found the following variables to be the most predictive: MDS-UPDRS Part I score, MDS-UPDRS Part II score and the MDS-UPDRS axial motor score. The combination of these predictors generalized well to the test dataset –overall accuracy and the area under the receiver-operating characteristics curve were 78.5% and 76.4%, respectively (see ROC curve in Fig. 1).

### Binary logistic model at 5-year follow-up and nomogram development

A multivariate logistic regression model was used to calculate the probability of functional dependence depending on the four variables found significant in the lasso model.

**Fig. 2:**
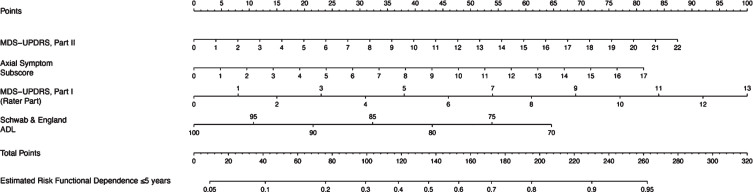
Nomogram for the prediction of functional dependence within 5 years. MDS-UPDRS, Movement Disorder Society-Unified Parkinson’s Disease Rating Scale; ADL, Activities of Daily Living. A nomogram model to predict functional dependence in early PD patients. Each associated factor is given a point value (total 100 points) which translates to a prognostic percentage of the likelihood that a patient will become functionally dependent within 5 years of diagnosis.

**Fig. 3:**
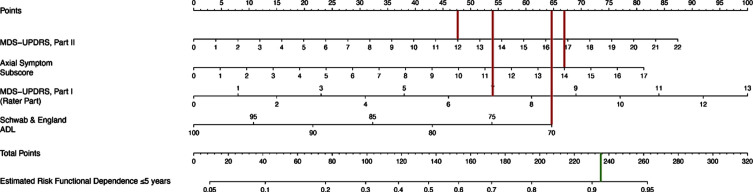
Example Nomogram. To read the nomogram, a vertical line is drawn for each variable of the patient, from the variable scale to the points scale. Then, the four values from the points scale are summed up to obtain total points. Finally, a vertical line is drawn from the total points scale to estimate risk of functional dependence within five years of PD diagnosis. For example, a patient with an MDS-UPDRS II score of 12, axial subscore of 14, MDS-UPDRS Part I score of 7 and Schwab & England score of 70 at the baseline visit would have a 91% estimated risk of becoming functionally dependent within 5 years of diagnosis.

Table 2 shows that functional dependence was associated with higher baseline MDS-UPDRS I score, per one point increase (OR 1.20, *p* = 0.021), higher baseline MDS-UPDRS II (OR = 1.10, *p* = 0.006), higher baseline axial score, per one point increase (OR = 1.20, *p* = 0.033) and lower baseline Schwab & England ADL scores, per one point increase (OR 0.95, *p* = 0.044). The corresponding nomogram is presented in Fig. 2. Figure 3 shows an example nomogram for a PD patient. To read the nomogram, a vertical line is drawn for each variable of the patient, from the variable scale to the points scale. An MDS-UPDRS score of 12 would equal 47.5 points, axial subscore of 14 equals 67, MDS-UPDRS Part I score of 7 equals 54 points and Schwab & England score of 70 equals 64.5 points. Then, the four point values from the points scale are summed up to obtain total points (233 points). Finally, a vertical line is drawn from the total points scale to estimate risk of functional dependence within five years of PD diagnosis (91%).

**Table 2:** Binary Logistic model

Characteristic	OR	95% CI	*p*
**Baseline MDS-UPDRS I**	1.20	1.03, 1.40	0.021
**Baseline MDS-UPDRS II**	1.10	1.03, 1.17	0.006
**Baseline MDS-UPDRS axial**	1.20	1.03, 1.40	0.033
**Baseline S&E**	0.95	0.91, 1.00	0.044

OR, odds ratio; CI, confidence Interval; MDS-UPDRS, Movement Disorder Society- Unified Parkinson’s Disease Rating Scale; S&E, Schwab & England.

## DISCUSSION

In this study, our goal was to explore predictors of functional dependence with regards to the PPMI dataset. We observed a deterioration of functional dependence for ADL in a cohort of 395 *de novo* PD patients after a 5-year follow-up. Besides the axial motor score, we identified MDS-UPDRS part I score and the MDS-UPDRS part II score as predictors of dependence using a nomogram.

The number of functionally dependent subjects in our study at any point within the 5 years was 85 (22%), this frequency is in line with other studies. Inception studies have reported the prevalence of dependency to be between 10–25% at 5 years and 20–50% at 10 years [2, 3, 7, 22]. Our results concur with others that the axial motor score is a predictor for the decrease of functional dependence [2, 4, 23]. Axial symptoms have a propensity to become resistant to dopamine replacement and device-related therapies [20, 25] and are therefore a particularly strong predictor of functional dependence. Indeed, axial motor deterioration can lead to reduced mobility and to various injuries through recurrent falls, rendering this sub-score effective in the use of this nomogram. Patients with the postural instability-gait difficulty phenotype (involving mostly axial motor impairment) experience cognitive problems earlier and have a steeper and faster decline [26, 27]. MDS-UPDRS part II was also shown to be a significant prognostic factor for functional dependence. This concurs with other studies, which have demonstrated concurrent, convergent, and predictive validity of UPDRS part II-based algorithms to define functional dependence in PD [28]. We found UPDRS part I rater section to be a significant predictor of functional dependence. The individual items in this section such as cognition, depression and anxiety are factors associated with the diffuse/malignant form of PD, identified to be a more rapidly progressing type of PD. Apart from more severe cognitive deficits and neuropsychiatric symptoms such as anxiety and depression at baseline, these symptoms also progress faster in these patients [29–31].

The nomogram from the present study provides an accurate visual tool to medical staff, caregivers, and older adults for prediction, early intervention, and graded management of PD. Prognostic models like nomograms may serve as a stepping stone in the growing area of personalized medicine to provide individual risk predictions. For example, a PD patient with an MDS-UPDRS II score of 12, axial subscore of 14, MDS-UPDRS Part I score of 7 and Schwab & England score of 70 at the baseline visit would have a 91% estimated risk of becoming functionally dependent within 5 years of diagnosis. Knowledge of prognostic indicators could be used to enhance the design of clinical drug trials, for example to stratify randomization, or as factors to adjust for in the analysis of observational studies. Nomograms may also prove to be helpful in grouping patients with similar prognosis together who may profit from the same treatment. Patients at risk to become physically dependent in 5 years may also be personally motivated to participate more in their own health journey and to remain ‘in the know’ about what they can do to combat future disease-related impediments.

Comorbidity, disease duration and cognitive issues have been associated with functional dependence. However, in these studies the baseline visit was often years after diagnosis of PD [4, 7, 8, 23]. The PPMI study on the other hand, enrolled patients at the very beginning of their disease. Age and bradykinesia were the only identified prognostic factors for functional dependence in a recent review [7]. Here, the patient population had quite a young age range (median: 63 years old, IQR: 55-70) at baseline, whereas in other studies, the mean age at diagnosis was in the late 60 s or 70 s [2, 4–6, 8]. The bradykinesia sub-score did not influence dependency in our calculations. In contrast to a previous report [22], probable RBD and the use of the RBD questionnaire here was not a predictor for functional dependence.

PPMI is an optimal tool for the assessment of the progression of PD through nomograms because of its longitudinal nature and annual visits; however, some scales like Physical Activity Scale for the Elderly, FOG-Q, and Falls Questionnaire were not administered at baseline, therefore, we were not able to check whether these had any predictive value. The advantage of the nomogram is a prognosis of individualized risk based on certain patient and disease characteristics, that it can better estimate disease course than a clinician, and its ease of use. The primary disadvantage is the assumption of nomograms that outcomes remain constant over time and that most of them lack external validity. Developing a nomogram based on PPMI data may have selection bias, however the fact that the project is a worldwide multicenter design, increases its external validity. Lastly, it should be noted that the AUC for this nomogram was mediocre therefore, there is a potential for distress or false reassurance by providing estimated risks to patients. It is important to conduct further studies to be able to accurately employ this nomogram in a real-world setting.

### Conclusion

Here, we delivered an easy-to-use tool in the form of a nomogram incorporating various parts of the MDS-UPDRS to predict the probability of functional dependence of a patient 5 years after the diagnosis of PD. Significant aspects were UPDRS Part I, II, and the axial motor score. Further research is necessary to include genetic, imaging, and other clinical rating scales as a way to create an integrated nomogram for the prediction of milestones in the loss of functional dependence.
